# The light side of gaming: creativity and brain plasticity

**DOI:** 10.3389/fnhum.2023.1280989

**Published:** 2024-01-05

**Authors:** Christiane Ganter-Argast, Marc Schipper, Manouchehr Shamsrizi, Christian Stein, Radwa Khalil

**Affiliations:** ^1^Department of Psychosomatic Medicine and Psychotherapy, University Hospital and Faculty of Medicine, University of Tübingen, Tübingen, Germany; ^2^University of Applied Sciences, Nürtingen-Geislingen, Nürtingen, Germany; ^3^University of Applied Sciences and Arts, Ottersberg, Germany; ^4^Institute for Psychology, Arts, and Society, Bremen, Germany; ^5^IFA – Institut für Auslandsbeziehungen, Stuttgart, Germany; ^6^Excellence Cluster Matters of Activity / Gamelab.Berlin, Humboldt-Universität zu Berlin, Berlin, Germany; ^7^School of Business, Social, and Decision Sciences, Constructor University, Bremen, Germany

**Keywords:** art therapy, brain plasticity, creativity, executive functions, flow, game, anxiety

## Abstract

Could gaming enhance brain plasticity and executive functions (EFs) by fostering creativity? We identify vital benefits from further research exploring the relationship between games, brain plasticity, and creativity. The ongoing progress in neuroscience research in these three disciplines offers many possibilities and prospects for impactful therapy. Therefore, we emphasize the significance of investigating the untapped potentials of using games in creative therapy—our perspective on the often-overlooked neuroscientific aspect of creativity concerning health and wellbeing. One of these potentials is examining games as a therapeutic tool, focusing on their capacity to inspire and engage the imagination and other mental operators shared with creativity. Using a game as a therapeutic approach may boost brain plasticity, which may help them reduce their cognitive impairments by improving their EFs. This review offers a comprehensive outline of the latest advancements in the literature on games that tie to creativity through enhancing brain plasticity and EFs. Communicating this knowledge can furnish countless possibilities to improve our overall health and wellbeing and foster a positive perspective in individuals affected by anxiety.

## Introduction

In 2019, WHO reviewed more than 900 studies on the impact of the arts on health and wellbeing, all demonstrating positive outcomes (Behavioural and Cultural Insights [BIF], 2019). Accordingly, it is imperative to call for direction toward effective creative therapy. Therefore, it is imperative to advocate for the greater use of creative therapy that considers the brain’s functions and process-based approaches. According to Polner et al. (2015), Guseva (2019), and Khalil and Demarin (2023), the brain’s extraordinary ability to adapt and change serves as an example of the promise of creative therapy in the arts. After thoroughly evaluating numerous theories on the conceptualization of creativity, games, and brain plasticity, we briefly present our perspective in this review and highlight how integrating disciplines could benefit effective therapy.

The multidimensional nature of creativity makes it challenging to define; it encompasses music, literature, art, movement, science, etc., which interact and interdepend, emphasizing the diverse perspectives involved (Abraham, 2013, 2018; Khalil et al., 2019; Khalil and Moustafa, 2022; Khalil and Demarin, 2023). In contemporary times, there has been a shift in the physiological approach toward comprehending creativity, with a focus on delineating its mechanisms within the framework of brain networks (Jung et al., 2013; Abraham, 2014, 2018; Beaty et al., 2015, 2016a,^2018^, ^2019^; Beaty and Schacter, 2017; Corazza et al., 2022; Khalil and Demarin, 2023) and learning rules^1^ (Khalil and Moustafa, 2022).

Many neuroscience studies on creative thinking focus on the central nervous system, particularly the brain. Few studies, however, have investigated the connection between creative engagement and peripheral nervous system function. Examples of this kind of research include the findings of increased sympathetic cardiac activity during creative divergent thinking, as reported by Silvia et al. (2014) and Khalil et al. (2023), and enhanced pupil dilation during music-induced aesthetic “chills” (Laeng et al., 2016). This research field, which investigates the impact of emotional regulation on creativity, merits further attention and examination.

On the flip side, the development of multiple-factor models of creativity, as creative mental operations, arose to expand upon the existing dual models. These multiple-factor models consider the simultaneous functioning of three or more systems (Abraham, 2018; Jung and Vartanian, 2018; Khalil et al., 2019; Ivcevic et al., 2023; Khalil and Demarin, 2023). The coordination of various operations of creative cognition is evident in the integrated dynamic that emerges during creative activities across various domains of human endeavor (Abraham and Windmann, 2007; Abraham, 2013, 2014; Khalil et al., 2019; Corazza et al., 2022; Khalil and Moustafa, 2022; Khalil and Demarin, 2023). The interaction between the two brain hemispheres through specific nodes, known as network hubs (Jung-Beeman et al., 2004; Jung et al., 2010, 2013; Beaty et al., 2015, 2016a,^2019^; Jung and Vartanian, 2018), explains one dimension of these dynamics. It has been suggested that these integrated dynamics have an impact on the mental workspace,^2^ including both deliberate and spontaneous modes, and the overall flow of experience (Dietrich, 2004, 2019a; Dietrich and Kanso, 2010; Saggar et al., 2015, 2017; Xie et al., 2021; Khalil and Demarin, 2023).

We have encountered situations where we faced challenges and found ourselves unable to make further progress, ultimately reaching a point of resolution. At this stage, individuals may experience some level of frustration but may also become aware of a potential solution (Sapp, 1992; Agnoli et al., 2019; Khalil et al., 2023). This insight phenomenon is often described as a sudden realization or moment of clarity, commonly referred to as an “aha” moment or “eureka” moment (Kounios and Beeman, 2009, 2014). This experience has facilitated the integration of new components into existing conceptual knowledge structures (McCrae and Costa, 1997; Ivcevic and Brackett, 2015; Li et al., 2015; Beaty et al., 2016b). When a solution is reached, it is possible for new insights to emerge naturally by overcoming functional fixedness and embracing a new perspective (Csikszentmihalyi, 1997, 2008; Dietrich, 2004; Gray et al., 2019; van der Linden et al., 2021), thus reshaping the mental workspace.

Games can boost brain capacity by fostering creative expression and cognitive growth (Anguera et al., 2013; Kühn et al., 2014; van Dijk and De Dreu, 2021; Abbott et al., 2022; Kleschnitzki et al., 2022). Digital games can dynamically adapt the environment to the player’s abilities, optimizing creativity (Roca et al., 2018); hence, games are player-centered rather than simulations (Narayanasamy et al., 2006). In the context of simulations, it is typically the case that the player is required to adapt to the simulation rather than the other way around. This is particularly evident in simulations involving flight or walking. Numerous player-centered methodologies employ this technique, leading to significantly enhanced immersive encounters demonstrating considerable therapeutic potential.

Games might be considered an artificial environment that prioritizes the player’s pleasure over accurately mimicking the real world. While uncommon or harmful behavior in real life is likely to have long-term, possibly negative effects, games provide a safe area for experimenting. These characteristics create an environment suitable for game creativity; the possible interactions within the games determine the range of mental abilities that can be nurtured. Different games combine strategic, expressive, reactive, or social elements, each with a distinctive blend and emphasis. Therefore, choosing a game that complements the particular player’s present abilities and passions is critical, allowing for more remarkable growth or exploration of new mental spaces (Anguera et al., 2013; Kühn et al., 2014; van Dijk and De Dreu, 2021; Abbott et al., 2022; Kleschnitzki et al., 2022). Various games tailored to different demographics and preferences enable the development of customized ludic interventions that stimulate creative processes, allowing individuals to path along uncharted routes and augment the brain’s plasticity. An example is using specially developed and commercially available games that positively impact plasticity.

The following section will address the connection between creativity, imagination, and mental workspace as examples of creative mental operators. Afterward, we will highlight the potential benefits of gaming in promoting brain plasticity and facilitating the flow of experience through creative art therapy. A patient case study demonstrating the effectiveness of creative art therapy will be emphasized, and a summary will be given.

## Creativity, imagination, and mental workspace

What factors contribute to the expression of exceptional creative behaviors exhibited by scientists, artists, musicians, dancers, and others? Psychological researchers have investigated various mental processes connected to creativity, including conceptual expansion, creative imagery, overcoming knowledge restrictions, analogical reasoning, and metaphor processing^3^ (Ward et al., 1995, 1997; Finke, 1996). A fundamental component of creative thinking involves conceptual expansion, which refers to broadening our perspectives and exploring beyond the limitations of our existing conceptual frameworks of semantic knowledge.

Essential abstract aspects, such as creative imagery, are necessary to generate original and beneficial ideas. Imaginary worlds have the potential to leverage our innate inclination for exploration, which is shared by both humans and animals, to guide individuals toward novel environments and sources of fulfillment (Dubourg and Baumard, 2022). Humans are drawn to fictional worlds for the same reasons and under similar circumstances that they are drawn to foreign surroundings in real life, assisting in overcoming abundant information challenges (Nettle, 2001; Abraham, 2016; Dubourg and Baumard, 2022). Modifying mental imagery is necessary for several creative and uniquely human capabilities; investigations conducted by Schlegel et al. (2013, 2016) utilized functional magnetic resonance imaging to study the cognitive processes associated with human mental imagery manipulation.

Therefore, imagination is among the crucial factors in enhancing creativity, serving as a particularly effective means of nurturing this process. Researching the origins of human imagination has been a topic of interest for philosophers and scientists over an extended period (Nettle, 2001; Abraham, 2016; Dubourg and Baumard, 2022). Many of the most widely embraced creations of fiction designed in recent decades feature a captivating fictional universe; they are commonly encountered in various forms of fictional media, including novels, films, and video games^4^ (i.e., The Legend of Zelda and Final Fantasy) and graphic novels (i.e., One Piece and Naruto). “Artists, civil society, and policymakers must engage in what is becoming a decisive moment in technology; [.] video games take on global significance for art, activism, and cultural diplomacy” (Fugleberg, 2023).

Games as “Gesamtkunstwerk”; the term “Gesamtkunstwerk” is a German concept that translates to “total work of art” or “universal artwork.” It refers to an artwork, design, or creative process in which various art disciplines are integrated to generate a single cohesive whole. This concept was popularized by composer Richard Wagner, who conceived his operas as a perfect union of artistic genres, from singing to orchestral music, dance, stagecraft, and acting. Similarly, video games can be seen as a modern form of “Gesamtkunstwerk” because they combine various forms of art and technology into one interactive experience; each element is crucial in creating the immersive worlds players explore in video games. This integration includes visual arts (graphics and design), storytelling (narrative and character development), music and sound design, architecture (in the design of game environments), and even elements of performance (through voice acting and motion capture).

**FIGURE 1:**
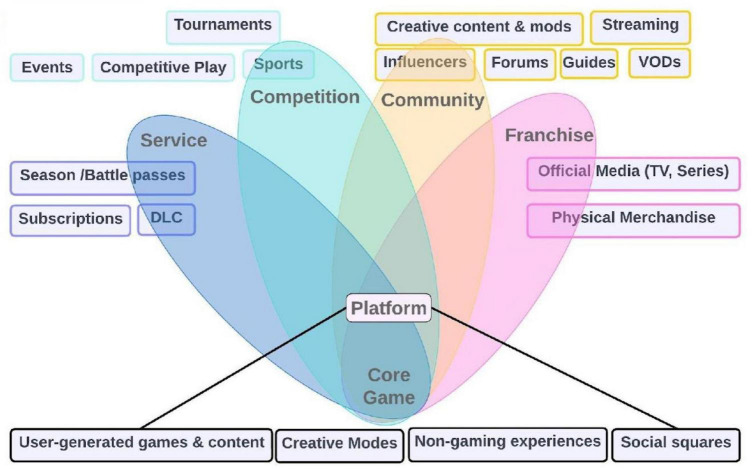
Illustration of the gaming metaverse. The bottom part represents the involved core game, which expands to the platform and the subsequent scopes: gaming as a service, competition, community, and franchise. The boxes depict examples of each scope.

To better understand the potential of this decisive moment for gaming in art therapy and beyond and why gaming’s impact goes way beyond entertainment, it is helpful to refer to a model of spheres, the “Gaming Metaverse,” widely recognized in the gaming community. This model is constructed in concentric circles around the actual game session (core session), which serves as the central point of departure. We represented this model using a 4-set Venn diagram (Figure 1).

The game is perceived as a platform where new content is generated far beyond the original art therapy session. This platform can be further developed and customized through the participation of patients and enthusiasts. The service scope can be utilized in an educational setting or to improve health-related knowledge and skills. The competition scope can be utilized for events, sports, competitive plays, and tournaments. The community scope includes influencers, forms, guides, steaming, creative content, and mods. The franchise scope is marked by pronounced transmediality; hence, the therapy extends beyond the traditional boundaries of art therapy. It intersects with other innovative industry sectors, for example, through books, video games, films, and TV series. A prime example is “The Witcher,” which initially emerged as a book series, then transformed into a video game, and ultimately evolved into a Netflix series.

Huili et al. (2023) developed and tested somatosensory interaction induced by games for autistic children, demonstrating the use of video games in art therapy. Moreover, the video game serves as an artistic intervention method employed for therapeutic purposes. The game consists of four categories: social interaction, emotional venting, cognitive education, and intellectual enhancement. The art team designed two somatosensory interactive themed games, one for puzzle-themed “Digital Maze” and the other for sports rehabilitation-themed “Happy Kitchen.” The somatosensory interactive games used in this study can also create virtual environments, which not only have better security and targeting but also have higher immersive potential. The game emphasizes feedback and reward mechanisms, such as positive affirmation of play and behavioral stimulation of positive emotions. It helps them practice their emotional regulation and social skills. A randomized scientific control found that the game group was more effective than the picture book group regarding physical interaction, facial expression response, and eye contact (Huili et al., 2023). Developing, creating, and using video games in art therapy is considered one possible “…form of technology-based media that therapists could use to support clients in creating art as part of the therapy process,” as Malchiodi (2011, p. 33) describes digital art therapy.

Regarding video games, they are not just a passive experience; they require active engagement from the player. This interactivity adds another layer to the “Gesamtkunstwerk” concept. Players are not just observers; they participate in the artwork, influencing its direction and outcome through their actions. Games offer an openness in which there is not necessarily a right, predefined solution but the possibility of finding a new one (Jackson et al., 2012).

As for the impact on creativity, studies have shown a positive correlation between video game playing and creativity. For instance, a study of nearly 500 12-year-olds revealed that the more children played video games, the more creative they were in drawing pictures and writing stories (Jackson et al., 2012). Another systematic review by Rahimi and Shute (2021) found that not all video games can enhance creativity—some game genres have more potential to enhance creativity than others. Specifically, video games that facilitate flow, allow the players to co-create the game, and enhance players’ intrinsic motivation have the most potential for enhancing creativity. Accordingly, video games can be seen as a form of “Gesamtkunstwerk,” combining various forms of art and technology into one interactive experience. This unique combination and the active engagement required from players make video games an excellent tool for fostering creativity.

A team led by Simone Kühn found influential gaming neuropsychological consequences by studying adults who played Super Mario 64 for 8 weeks for 30 min daily for video game effects (Kühn et al., 2014). The study by Kühn et al. (2014) demonstrated that a 2-month intervention with platform video gaming induces structural plasticity effects in the right hippocampus (HC), right dorsolateral prefrontal cortex (DLPFC), and bilateral cerebellum. The trained subjects experienced a volumetric increase in the right HC, linked to a shift from egocentric to allocentric navigation. A positive correlation was observed between the participants’ weekly ratings of their desire to play video games and increased gray matter in HC and DLPFC. The association between the reported desire and the DLPFC’s growth was more robust in the first month compared to the second. Therefore, the reported desires might cause DLPFC growth rather than DLPFC growth, causing an increase in desire.

However, future research must rectify the limitations of this study by Kühn et al. (2014). Firstly, prospective studies on video game training should incorporate an active control group that receives novel technological equipment to investigate, mirroring the training group. The advantage of that is the exclusion of the likelihood that the observed training benefits result from examining the new equipment rather than the actual gameplay. The results suggest that the specific game genre used in this study may contribute to the targeted development of HC and DLPFC. Future research should conduct experiments with various game genres to illustrate the distinctiveness of structural plasticity in the HC for video games that involve navigation. Future research should conduct experiments with various game genres to illustrate the distinctiveness of structural plasticity in the HC for video games that involve navigation. For instance, an earlier study by Haier et al. (2009) examined the structural impacts of video game training, specifically focusing on the game Tetris. Haier et al. (2009) studied cortical thickness measures precisely and did not observe elevation in DLPFC or HC; nevertheless, they did observe an increase in the frontal eye fields and the temporal pole.

Similarly, it would be beneficial to have a more accurate record of gaming achievements to link observed alterations in structure with performance metrics. Lastly, forthcoming research in this domain should use comprehensive test batteries to assess a broader range of cognitive functions the game will likely improve, such as executive functions (EFs).^5^

To build on these findings, a new initiative at Humboldt-Universität’s game lab, Charité, Berlin, and the second largest public health insurance company, BARMER, conducted a study that revealed the positive impacts of a gaming-based intervention on the cognitive abilities of seniors. The study encountered that regular engagement with the serious game MemoreBox had a noticeable impact (Kleschnitzki et al., 2022). The findings suggest that incorporating an accessible serious game into the standard care provided in nursing homes could be a valuable addition to the healthcare system; this is especially important given the recognized need for more engaging activities for senior citizens in partially inpatient care facilities (Kleschnitzki et al., 2022). The findings of this study indicate that the intervention had a significant effect on the cognitive capabilities of older adults, given their consistent engagement with the serious game called “MemoreBox.” Moreover, integrating a user-friendly serious game into the standard care provided in nursing homes could potentially address the healthcare system’s shortcomings, particularly the limited availability of stimulating activities for seniors residing in partially inpatient care facilities (Kleschnitzki et al., 2022).

## The power of gaming: augmenting neuroplasticity through creative art therapy

Games have the potential to positively impact one’s mental capacity by providing opportunities for creative expression and cognitive growth (Anguera et al., 2013; Kühn et al., 2014; van Dijk and De Dreu, 2021; Abbott et al., 2022; Kleschnitzki et al., 2022). While real-world challenges may occasionally count on the chance to find the ideal balance between difficulty and players’ abilities, games strive to consistently achieve and maintain this balance as much as possible. This balance usually creates the most motivation for the player to continue and increase his capabilities. Simultaneously, games promote a constant improvement of players’ abilities rather than cultivating a mindset centered primarily on the passive consumption of the medium. Therefore, the game’s difficulty level is intentionally crafted to correspond with the anticipated learning progression of the player. Adaptive difficulty levels are tailored to accommodate individual learning rates and trajectories (Sampayo-Vargas et al., 2013).

**FIGURE 2:**
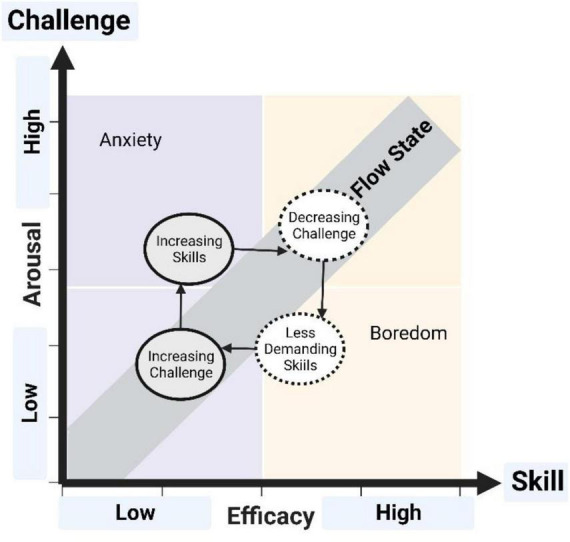
Flow state in games after Csíkszentmihályi (1990).

The concept of “flow” refers to a feeling of fulfillment that involves becoming unaware of time during an intense moment, i.e., when an individual focuses on an activity that matches their competency level and task demands (Csikszentmihalyi, 1997, 2008). As the skills increase through play, the difficulty should also increase to keep the player in the flow channel and avoid boredom or anxiety (Figure 2).

Most studies on the phenomenon of flow experience have primarily focused on physical activities involving bodily movement, such as sports, exercise, and dance (Jackson and Eklund, 2002). Facilitating the flow experience necessitates implicit and unconscious information processing across brain systems through transient hypofrontality^6^ (Dietrich, 2004, 2019a,b; Chrysikou et al., 2014; Chrysikou, 2018, 2019).

In the context of games, setting the flow status is a prerequisite; digital games, in particular, have the unique ability to adapt the environment to the player’s abilities dynamically, which can effectively optimize the conditions for the emergence of creativity (Roca et al., 2018). Games are not simulations but are entirely focused on the player’s experience (Narayanasamy et al., 2006). Therefore, games can be considered an artificial environment that prioritizes the player’s experience rather than perfectly replicating the real world.

Fascinatingly, games establish feedback loops that provide immediate responses to players’ activities and tend to be lenient, implying that they enable players to make subsequent attempts after unsuccessful endeavors (Kühn et al., 2014; van Dijk and De Dreu, 2021; Kleschnitzki et al., 2022). Therefore, games foster an increased propensity for individuals to take risks and participate in experimental endeavors, as they reduce the potential ramifications. The game’s safe environment facilitates an enhanced perception of autonomy and an incentive to engage in novel strategic approaches. While in real life, unconventional or unruly actions are likely to lead to long-lasting, potentially negative consequences, games open a free space for experimentation. This is exactly what can be used therapeutically to find new perspectives and solutions.

These attributes lead to establishing an environment suitable for fostering game creativity; the available interactions within the games influence the range of mental abilities that can be cultivated.

Creativity can be measured by the risks players take and the solutions they find. The more open a game is to different approaches, combinations, and interactions, the more creative potential it offers. Solving a riddle, for example, forces the player to recognize a series of hints and draw the expected conclusions correctly, thus creating a predefined problem-solving pathway. In contrast, a game like Backgammon instead opens a space of creative possibilities, which are not essentially right or wrong but equally possible alternatives. In many open games, players develop strategies that game designers have never considered. In that sense, creativity can be measured by analyzing brain activity and structure directly in the game by observing the variety of approaches to an open problem. Player actions can be logged and compared, and players with especially unconventional approaches can be interviewed later. Integrating this kind of logging into the game can offer exciting insights for developers and researchers alike, triggering creativity and individual strategies to develop new solutions that may be identified and integrated into future creativity-fostering game developments.

Various games incorporate diverse strategic, expressive, reactive, or social elements, each with its unique combination and emphasis. Therefore, selecting a game that aligns with the individual player’s existing skills and interests is of utmost priority, allowing for further development or exploring new mental spaces. Various games catering to different demographics and preferences make it feasible to develop customized ludic interventions that foster creative processes, enabling individuals to venture into unexplored paths and augment the brain’s plasticity.^7^

An example is using specially developed games as much as commercially available games with positive side effects on brain plasticity. Winnicott (1971) argues in one of the most famous phrases in psychoanalysis that “playing is itself a therapy.” If we want to take his statement seriously in contemporary psychotherapy, we should give the video game more meaning. Art therapists receive training in playing with their clients, assisting them in creating their own visual worlds that express their feelings and suffering, and seeking ways to cope with them through creative activity. Video games are, therefore, another artistic medium of expression with which this seems possible. However, art therapy is only beginning to use digital technologies such as video games for new, contemporary forms of intervention, particularly to better reach young people or people with limited mobility. Video games are just as enjoyable for online-based art therapy as for home treatment (Ganter-Argast and Bocksch, 2023). Moreover, individuals with illnesses such as myalgic encephalomyelitis and chronic fatigue syndrome, as well as prisoners, can also experience external realities within their living environment through video games.

On the one hand, for patients with high self-criticism, art therapy outcomes can also be seen as expressions of skill compared to others (Rankanen, 2014). Games, on the other hand, are in far more unmarked spaces. Playing a game is more about individually developing a skill and the process of creating it than about a finished product. Therefore, integrating games into art therapy can have a liberating character for the players and allow for more expressivity. When there is less comparison of the outcome with other works, it allows for a greater focus on individual expression.

As highlighted by Ibanez and Zimmer (2023) and Khalil and Demarin (2023), the brain’s plasticity and metaplasticity play a crucial role in creative therapy. The induction of brain plasticity, which can lead to improvements in EFs,^8^ has been associated with various factors, including musical training, improvisation, and exercises. The effectiveness of these factors is influenced by their duration and intensity, which can contribute to short-term and long-term improvements. Therefore, understanding the mechanisms that contribute to the promotion of creative outputs through the concept of neural plasticity is still a scope that demands further investigation.

The extension of many experimental paradigms (such as behavioral research and experimental psychology) to neuroscientific frameworks is even more challenging when studying creativity, which is complex and subjective (Abraham, 2018; Khalil et al., 2019). The challenge shows up in high creative task variability (allowing the assessment of creative thinking) (Arden et al., 2010) or in the problem of validity (e.g., the inability to reliably exogenously or endogenously prompt creativity). Here, gaming may serve as a new tool for dealing with these problems, for instance, by directly investigating the joint operations of creative cognition (Fink et al., 2009).

Therefore, by evaluating the cognitive processes that promote creativity through games from the plasticity perspective, we can gain valuable insights into these fascinating abilities and the unique neural pathways that support this remarkable level of adaptability.

The following section will discuss approaches to enhancing patient creativity and evaluate the potential benefits of incorporating gaming into a compelling (creative) therapy. Few studies have examined the therapeutic potential of tabletop role-playing games for social anxiety and other mental illnesses. An example of this study was the study by Abbott et al. (2022), which described a year-long Dungeons and Dragons therapy approach, evaluating the opinions of the group’s participants, the model’s basic concepts, and the developers’ lessons for therapists interested in using this model. Participants were more confident in social situations, especially when setting boundaries or navigating potential errors; thus, the game’s skills were also beneficial in real life.

## A case study of enriching the creative state of mind: games/gaming in (creative) therapy

Gaming, both as an industry and as a community of users, has transmedial impacts on arts and culture beyond the cultural technique of gaming itself. As an example, many of today’s “proto-metaverses”^9^ —Usually, the triad of “Roblox,” “Minecraft,” and “Fortnite” are categorized as early forms of gaming-based “metaverses” that empower their players for creative expression and exchange. When delving into the rich and diverse history of the metaverse, one can find many approaches that were developed even earlier than those mentioned above (Lawton, 2022). Therefore, categorizing them as early approaches does not hold, but they represent essential steps regarding the development of the metaverse.

In recent years, virtual concerts and art exhibitions have collaborated, i.e., with the Serpentine Gallery.^10^ In this light, European cultural policy is picking up on gaming, as exemplified by French President Emmanuel Macron, who in 2022 argued that “the metaverse has enormous potential for culture and leisure, thanks to its applications in music, concerts, art exhibitions, etc. we cannot consider our cultural policy without this revolution.”

A detailed evaluation of the game research in numerous domains, such as neuroscience, behavioral ecology, environmental aesthetics, and evolutionary and developmental psychology, reveals that various reasons cause differences in exploratory preferences (Kleschnitzki et al., 2022). These elements, which fluctuate between time and geographical locales, may assist in explaining variations in cultural preferences for imaginative worlds. As a result, this rationale explains the cultural evolution, shape, and substance of imagined worlds alongside their recent remarkable successes and broad presence across time periods and populations (Kleschnitzki et al., 2022).

Creation is essential to learning because it allows us to build new synaptic connections, which support mental health interventions like cognitive behavioral therapy. Systems theory provides insights into creative and artistic design processes, which may be shown in the success of art therapy (Pfab, 2018). It is necessary to assess their novelty or originality level, usefulness or efficacy, and incorporate aspects of surprise and unpredictability to categorize processes or systems as creative. As a result, the creative act primarily entails acknowledging and appreciating creativity; it is recognized that creativity is not a constant quality of a state of affairs or object but rather a variable value (i.e., a dynamical feature). For example, creativity can emerge from a natural shift in awareness, often prompted by an external event (such as a coincidence) in the surrounding environment, promoting a thoughtful appraisal of the transformational experience.

**FIGURE 3:**
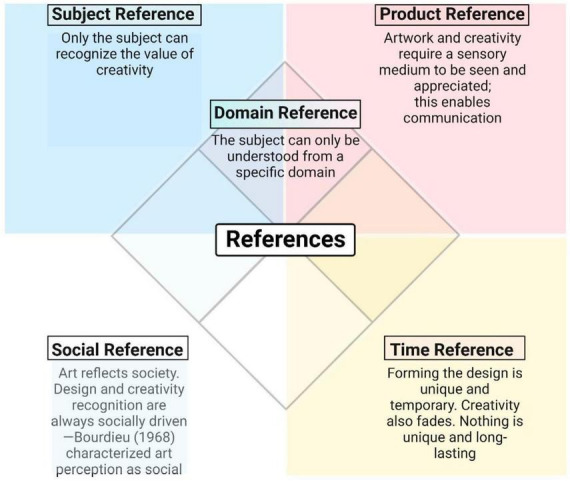
A graph showcasing the two primary attributes of novelty and utility, contingent upon five reference conditions of creativity that align during the artistic design process, encompassing analog and digital mediums. The subject reference indicates that artistic process and originality depend on a thinking subject, and only the subject can recognize creativity’s worth. Product reference relates to artwork, and creativity requires a perceptually perceivable medium on which they can be viewed and deemed significant; this facilitates communication. Domain reference means content that can only be comprehended and worked on from the perspective of a specific domain. Social reference is a reflection of society in works of art. Social factors influence the design process and the capacity to recognize creativity. Previously, in 1968, Bourdieu characterized art perception as a profoundly sociological phenomenon. Forming the design is a unique and transitory aspect of time reference; creativity is similarly impermanent. Nothing can be novel and useful over time, which refers to the time reference. The concept of novelty and usefulness is contingent upon the passage of time.

Pfab (2018) observed that when a participant makes a new use of an object that could be used in different ways, i.e., having the potential to be appropriately placed in new contexts, the creative outcome is not just the object itself but also the recognition and evaluation of its potential. This applies to physical and virtual objects, allowing for a wide range of digital experiences. Two fundamental characteristics of novelty and utility depend on five reference conditions of creativity that converge in the (analog and digital) artistic design process (Figure 3).

**FIGURE 4:**
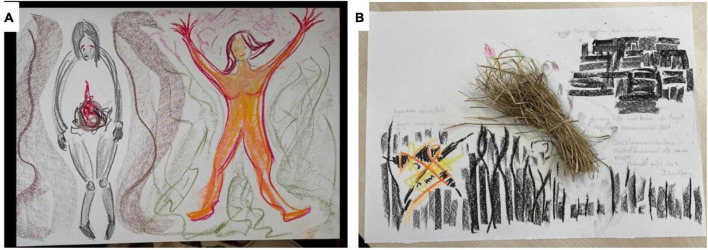
A depiction of a case study evaluating the application of art therapy within the mental health discipline. Using a brief movement exercise and innovative implementation, a patient with a recurrent depressive disorder has the following unexpected and novel experience: The movement exercise invites the patient to initially perceive her current posture and trace it, naming or describing it. Then, a movement impulse can be administered or relieved. This second movement may emerge spontaneously or through experimentation. It can also be given a name and intensify over time. As shown in Panel **(A)**, the experience can be expressed creatively. Panel **(B)** describes a novel experience conveyed creatively through a brief movement exercise. This mental creative process reflected the current mental state and initially elicited resignation and frustration. The design stagnated as helplessness spread (“maybe the ribbon will tear”) and hindered.

In 1954, Carl Rogers beautifully outlined conditions of constructive creativity that hold significant relevance in art therapy. This example demonstrates that these conditions are widely recognized as essential prerequisites for effective art therapy practice. Creativity can flourish when there is a willingness to consider and embrace the client’s unique concepts, beliefs, and perceptions (Rogers, 1954). In the opinions of Rogers (1954) and Pfab (2018), it is emphasized that recognizing the value of creating or creativity lies solely with the individual. Possessing the capacity to engage in playful and spontaneous experimentation is vital to gaining a new perspective on life. Rogers suggests that creative performance can sometimes arise from a feeling of solitude and that creativity serves to share and reconnect with others; thus, promoting safety, appreciation, and freedom fosters creativity. An atmosphere that encourages creativity involves a lack of external evaluation and instead fosters a sense of understanding and complete freedom in symbolic expression (Rogers, 1954). Recent virtual reality (VR) research advancements have created devices that generate immersive atmospheres. It is worth noting that this approach naturally provides all the necessary “mechanisms” that a gamification-guided intervention design may require; this could enhance analog creative therapy by creating a user-specific app. As a result, this app would encourage desired behaviors and deliver therapy-aligned, engaging tasks. Presented below are two illustrations of the practical effects of art therapy. The first example is the patient who reports her first slumped posture, full of suffering, especially in the abdominal area (a “burning” pain, Figure 4A).

The second movement impulse, intuitively accompanied by large space-occupying, circling arm movements, enabled the surprising experience that she automatically managed to free herself from this first position and to feel a positive change in herself in the short term. She was more accessible, full of energy, and carrying the fire to the outside (Figure 4A). She says, “I really have to do that (letting out fire or anger) sometimes!” Observing her being able to act again by creating or moving spontaneously gives her hope to regain more control and influence in other areas of her life. So, she could see her unexpected creative impulse to move as helpful in another context. The drawing itself again intensified the emotional levels and allowed the movement experiences to be relived. In addition, the picture made it possible to visualize, communicate, reflect, and recognize one’s creative production.

Another example from an art-therapeutic complement shows how incorporating natural materials enhances imagination. In moments of surprise, individuals may draw metaphorical connections between familiar natural objects and their circumstances. Like the previous example, the patient opts for a gently secured, dried tuft of grass from the assortment of natural objects available (Figure 4B). She quickly links this dried tuft of grass to missed chances and her present emotional condition. There may be some concerns about the stability of this situation. The delicate balance of nature relies on the resilience of a slender blade of grass. She mentions that maintaining composure can be challenging and requires significant effort.

By creating a supportive and encouraging environment, the patient may feel empowered to explore experimental artistic activities to express a range of emotions, including anger and sadness, in their artwork. As a result, there is a recognition of either mitigating these effects or engaging in impulsive actions. One potential therapy goal that could be considered is developing affect regulation skills; an art therapy gaming app could be utilized to facilitate this process.

While creativity gathers more and more attention in a salutogenic sense nowadays, based on growing empirical research activities showing its high potential in clinical and social intervention, the repertoire of tools available for the implementation of creative environments also extends to artificial intelligence, gamification, serious gaming, and digital developments such as VR and augmented reality (AR), which opens a whole new range of creativity-based interventions. In essential psychotherapy, for instance, VR is already successfully used to treat specific phobias like arachnophobia or vertigo, e.g., virtual exposition therapy (Lindner et al., 2019).

**FIGURE 5:**
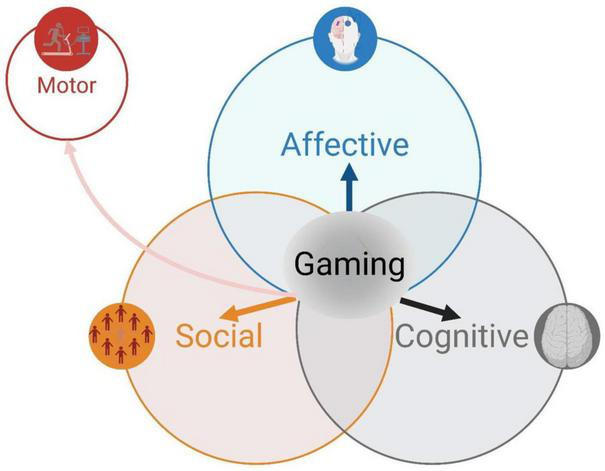
A diagram shows gaming as a multidimensional approach that effectively serves the bio-psycho-social model of health. Depending on the specific area of the focused domain, gaming can influence the social, cognitive, affective, and motor domains. As a result of frequent oversight, the motor domain is significantly far from the other three domains. Nevertheless, the motor domain is similar to the digital environment. For example, digital gaming makes it easy to include forced movements in the act of “playing,” such as by using kinetic sensors or VR/AR to control tasks through body responses.

Gamification and AI are coming into play by addressing clients’ motivation (in terms of generating adherence and compliance using APPs or DiGAs^11^) or adapting to their abilities, reducing frustrating experiences (Käser et al., 2013). One outstanding illustration of the promising applications of gaming in therapy, specifically digital creative therapy, is an innovative approach pioneered by Adam Gazzaley and his esteemed team at UCSF.^12^ They designed a tool that dynamically adapts to the environment, stimuli, challenges, and rewards in real-time, aligning with the individual’s brain activity. NeuroRacer, a video game developed by a collaborative team of neuroscientists, psychologists, and developers, was designed to create an immersive and visually appealing experience. This game operates as a closed-loop system, supporting the brain’s attentional processes (Anguera et al., 2013). Therefore, this game challenges neural networks, which are imperative for EFs, including resolving interference, switching tasks, and resisting distractions (Abbott, 2013; Schipper and Scherenberg, 2017). This approach experienced rigorous empirical testing and demonstrated its effectiveness, highlighting the considerable potential of creativity in clinical intervention and the numerous benefits of interdisciplinary collaborations. However, not only the cognitive domain but other domains represent a subject for gaming. In the following section, we provide further examples of its application in the whole range of the bio-psycho-social health model (Figure 5).

An example of the use and potential of gaming in the domain of social cognition is given by Dandeneau and Baldwin (2004) from the Social Cognition Lab at McGill University. In two studies, they examined the inhibition of rejection information. In the first study, the Rejection Stroop task measured an attentional bias toward rejection words hypothesized to characterize individuals with low self-esteem. These results indicated that people with low self-esteem experienced significantly more interference in rejection than in acceptance. In contrast, there was no such difference for people with high self-esteem. In a second study, a task was developed to train the response to inhibiting rejection information by repeatedly identifying the smiling or accepting face in a 4 × 4 matrix of frowning faces. These results showed that after this inhibition training, people with low chronic self-esteem experienced significantly less interference on rejection words on the Rejection Stroop than their counterparts in the control condition. On the other hand, people with high self-esteem did not exhibit different amounts of interference in rejection or acceptance words between conditions. These findings suggest that teaching people skills that help them deal with negative social information is possible.

Adaptive computing is highly relevant in the affective domain, as a study by Rauscher et al. (2016) showed. They demonstrated that the adaptive training program Calcularis can be used effectively to support children with developmental dyscalculia or math difficulties in their numerical development and to enhance numerical cognition while reducing math anxiety by adapting to the children’s strengths, down-powering frustrating experiences with arithmetics.

## Summary and concluding remarks

**FIGURE 6:**
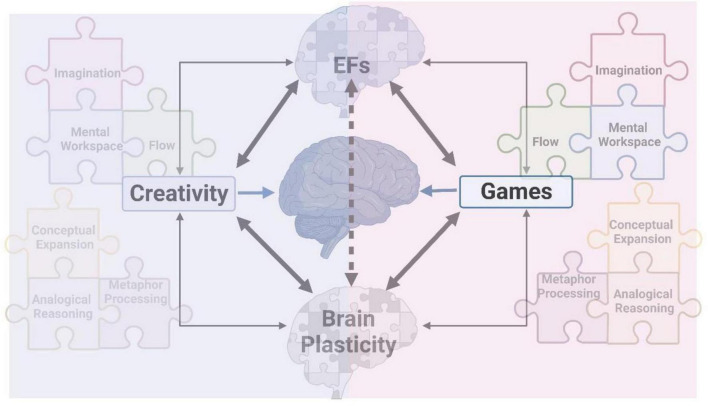
Description of creative art therapy using gaming to promote brain plasticity and strengthen executive functions (EFs). Creativity and games involve common mental operators, such as elements of imagination, mental workspace, flow, conceptual expansion, analogical reasoning, and metaphor processing.

Creative therapy has been shown to have the potential to significantly improve our overall state of wellness, providing a source of optimism. This review outlines our perspectives on neurobiological aspects of creative thinking and probes the potential effectiveness of creative therapy using games, emphasizing several vital issues that would benefit from further investigation to implement creative therapeutic approaches effectively (Figure 6).

This review reflects our perspectives on implementing effective creative therapeutic approaches in the context of game usage. It requires a comprehensive and cohesive understanding of how our current knowledge from the psychological and neuroscientific literature on creativity games could positively enhance EFs and brain plasticity. Therefore, providing empirical insights into the underlying neural mechanisms of new cognitive abilities that may be developed through games and creativity is beneficial. Games in this regard can be understood as tailored learning environments that combine cognitive development, creative freedom, and motivation.

Finally, it is crucial to recognize the limitations of manifold hypotheses that link game, creativity, and brain plasticity, instead considering creativity and gaming to suggest future research topics to prevent sweeping generalizations and ensure complete and accurate inquiry. As a result, creative therapy could be used as an effective therapeutic alternative.

## Author contributions

CG-A: Conceptualization, Writing – original draft. MSc: Conceptualization, Writing – original draft. MSh: Conceptualization, Writing – original draft. CS: Conceptualization, Writing – original draft. RK: Conceptualization, Writing – original draft, Visualization, Writing – review and editing.

## Footnotes

1 There is a plethora of activities that are associated with the process of learning and are known to engage different regions of the brain. To establish a more coherent relationship, researchers and mathematicians have created the concept of a learning rule, which pertains to enhancing the artificial neural network’s performance through its application throughout the network. The learning rule modifies a network’s weights and bias levels when specific criteria are satisfied throughout the training process. This component is deemed indispensable for the development of neural networks.

2 Mental workspace refers to a brain network responsible for deliberately manipulating images, symbols, concepts, and hypotheses, enabling individuals to address complex problems and generate creative ideas effectively.

3 Using innovative concepts across different domains, commonly referred to as analogical reasoning or metaphor processing, can be considered a promising method for enhancing our understanding of a phenomenon in diverse contexts (Mashal et al., 2007; Gold et al., 2012; Rutter et al., 2012).

4 Videogames are not adaptive; this is one prime factor of digital games (which many see as a supercategory of video games). One example of digital adaptive approaches in learning therapies is the software “Calcularis,” which adapts to the abilities of individuals, including those suffering from dyscalculia, to avoid frustrating or aversive experiences and reduce math anxiety.

5 EFs enable mental play, thinking before acting, confronting unexpected problems, resisting temptations, and staying concentrated (Diamond, 2013). Inhibition, working memory, and cognitive flexibility are core EFs.

6 Transient hypofrontality is currently the most widely accepted theory about the neural basis of the flow experience. It indicates that for a while, under certain conditions, the focused thinking part of our brain gets a rest. This permits other components and functions to take precedence.

7 The concept of “brain plasticity,” also known as “neuroplasticity” or “cortical mapping,” relates to the changes that can occur in the neural pathways of the brain at different levels, including macroscopic, mesoscopic, or microscopic (synaptic) levels. These changes can occur because of various factors, including modifications in behavior (such as those induced by training), responses to the environment (such as exposure to enriched or impoverished conditions), and physiological processes (such as maturation or brain insults). This phenomenon has been extensively studied by numerous scholars (Markram and Tsodyks, 1996; Tsodyks and Markram, 1997; Buonomano and Merzenich, 1998; Tsodyks et al., 1998; Abbott and Nelson, 2000; Blitz et al., 2004; Caporale and Dan, 2008; Citri and Malenka, 2008; Gerstner, 2011; Hamada et al., 2012; Carcea and Froemke, 2013; Froemke, 2015; Khalil et al., 2017a,b, 2018). Buonomano and Merzenich (1998) and Allen et al. (2003) imply that brain plasticity contradicts the idea that brain functions are unchangeable. Zavoreo et al. (2012) call the human brain “plastic” and “flexible” due to its extraordinary adaptability to various experiences.

8 EFs enable mental play, thinking before acting, confronting unexpected problems, resisting temptations, and staying concentrated (Diamond, 2013). Inhibition, working memory, and cognitive flexibility are core EFs.

9 As discussed elsewhere, a productive techno-philosophical working definition of “metaverse” could build up on Milgram and Kishino’s reality-virtuality continuum and the recent debate on co-sensing, such as Ophelia Deroy’s concept of “joint-perception.” Therefore, a gaming-based metaverse could be described as “an interactive environment in the reality-virtuality continuum that allows for real joint-perception.”

10
https://www.fastcompany.com/90713363/fortnites-first-ever-art-exhibit-is-also-a-kaws-experience-at-londons-serpentine-gallery

11 Digitale Gesundheitsanwendungen.

12 University of California San Francisco.
